# Pharmacopoea Generalis

**Published:** 1783

**Authors:** 


VI. *Pharmacopoea Generalis edita a D.* Jacobo Reinboldo Spielman. 4to. *Argentorati*, 1783. p. 646.

IN this work the learned Profeſſor Spielman has undertaken to bring together all the medicinal compoſitions that are in uſe in different countries. For this purpoſe he has had recourſe to a great variety of Diſpenſatories, and other medical writings of which the titles are enumerated in a *Syllabus Auctorum*, that extends through 15 pages.

In his Introduction he points out the beſt method of ſtudying and practiſing pharmacy, and explains the different weights and meaſures of the ancients and moderns, chemical characters, &c. The work itſelf is divided into two parts. In the firſt, or *Materies Pharmaceutica*, we have a very good deſcription of all the medicinal ſimples now in uſe. This part, the author informs us, is not copied from books, but founded on his own careful examination of the different articles. What he ſays of the Uva Urſi will ſerve as a ſpecimen of his method.

"Uva Ursi, *Bouſſerole*, *Steinbeere*. Folia "petiolata, ovata, oblonga, obtuſa, integerrima, "duriuſcula, ſupra viridia, rugoſa, ſubtus pal-

"lida,

"lida, inodora, cum quadam amaritie adſtrin-

" gentia. *Arbutus Uva Urſi* L. Fruticulus

" paſſim per Europam obvius. Folia dantur

" ſub forma pulveris ad drachmam unam, cum

" Aquæ Unciis duodecim decocta ad drachmas

" duas contra affectus nephriticos."

The ſecond part contains the compound medicines: and here the learned author confeſſes he has been obliged, from the nature of his plan, to give place to many uncouth remedies, which he would otherwiſe have gladly rejected. In a note at the end of each article, he mentions its virtues, and the doſes in which it is adminiſtered.

